# pH drop impacts differentially skin and gut microbiota of the Amazonian fish tambaqui (*Colossoma macropomum*)

**DOI:** 10.1038/srep32032

**Published:** 2016-08-18

**Authors:** François-Étienne Sylvain, Bachar Cheaib, Martin Llewellyn, Tiago Gabriel Correia, Daniel Barros Fagundes, Adalberto Luis Val, Nicolas Derome

**Affiliations:** 1 Institut de Biologie Intégrative et des Systèmes (IBIS), Département de Biologie, Université Laval, Québec, QC, G1V 0A6, Canada; 2 School of Life Sciences, University of Glasgow, Glasgow, G12 8QQ, United Kingdom; 3 Federal University of Amapá (UNIFAP), AP 68.903-419, Brazil; 4 Instituto Nacional de Pesquisas da Amazônia (INPA), Laboratório de Ecofisiologia e Evolução Molecular, Manaus, AM, 69067-375, Brazil

## Abstract

Aquatic organisms are increasingly exposed to lowering of environmental pH due to anthropogenic pressure (e.g. acid rain, acid mine drainages). Such acute variations trigger imbalance of fish-associated microbiota, which in turn favour opportunistic diseases. We used the tambaqui (*Colossoma macropomum*), an Amazonian fish tolerant to significant pH variation in its natural environment, to assess the response of fish endogenous microbiota to acute short-term acid stress. We exposed 36 specimens of tambaquis to acidic water (pH 4.0) over 2 consecutive weeks and sampled cutaneous mucus, feces and water at 0, 7 & 14 days. The 16S RNA hypervariable region V4 was sequenced on Illumina MiSeq. After two weeks of acidic exposure, fecal and skin microbiota taxonomic structures exhibited different patterns: skin microbiota was still exhibiting a significantly disturbed composition whereas fecal microbiota recovered a similar composition to control group, thus suggesting a stronger resilience capacity of the intestinal microbiota than cutaneous microbiota.

The bacteria composing the microbiota of eukaryotes have contributed to the evolution of metazoans by playing key roles in metabolic processes affecting fitness of their host^1^,^2^. Roles of endogenous microbiota have been documented in immunity^3^, growth^4^, mating preference^5^, behaviour^6^ and energy utilization^7^ of the host. The importance of endogenous microbiota in metazoan evolution is such that some suggest hosts and their microbiota are best understood as a single meta-organism; defined as the holobiont^8^. On teleostean hosts, endogenous symbiotic bacteria have been documented in the intestinal tract^9^,^10^,^11^, the cutaneous mucus^12^, and on the gill’s surface^2^. The microbiota found on fish body surfaces is the very first line of defence against opportunist pathogens from the environment^13^,^14^,^15^,^16^. The strength of this first line of defence (so called ‘colonisation resistance’^17^) may rely on the equilibrium between the relative abundance and diversity of different species of endogenous bacteria. Physiological stress of the host may promote proliferation of opportunistic pathogens via perturbation of the equilibrium between relative abundance of endogenous commensals (so called ‘dysbiosis’)^18^,^19^. A number of factors have been reported to drive dysbiosis in teleost-associated microbiota, including anoxia^16^, exposure to surface-acting disinfectants^20^ or inappropriate diet^21^. Variations in water physico-chemistry are likely to also drive microbial dysbiosis in fish given the permanent contact of fish with surrounding water in their environment.

In the last few years, there has been growing concern about the effect of pH decrease in water bodies^22^. Increased post-industrial concentrations of sulphuric and nitric oxides in the atmosphere decreases the pH of rain in urban areas^23^. After an acidic rainfall, the environment is temporally acidified. The resistance and resilience of ecosystems exposed to acidic rainfall relies on a multitude of abiotic and biotic characteristics from the environment: the presence of exogenous polyamines^24^, local soil chemistry^25^ or the ionic composition of the water, all of which potentially affecting the response of an ecosystem following acid precipitation. In this respect, watercourses with reduced carbonate hardness are particularly susceptible to pH drop when exposed to acid rain phenomenon, as they have a low buffering capacity.

The Amazon River is the largest drainage basin in the world^26^. The black water tributaries of the Amazon River have a naturally acidic pH combined with low carbonate hardness, and are thus highly sensitive to the effects of acid rain. The increasing anthropic pressure in Amazonia due to the large-scale urbanisation and industrialisation around and inside this ecosystem (e.g. the city of Manaus) could potentially affect the pH of water bodies in the near future. Freshwater capture fisheries in the Amazon represent a key component of Brazilian freshwater output (*248.8 Kilotons*)^27^. Thus, it is of major interest to assess the effect of water acidification on fish stock sustainability. Several species of Amazonian fishes are relevant models to assess the effect of acid stress, many of them being naturally exposed to two different types of water during their life cycle: white water (circumneutral pH) and black water (acidic pH ≈ 4.0–4.5). The physiological response of Amazonian fishes exposed to acid stress has been documented on the tambaqui (*Colossoma macropomum*), the most commercially important fish in Brazil, that migrates annually from white water to black water streams to feed during the rainy season^28^,^29^. Tambaqui exposed to acid stress experience an increase in plasma glucose, cortisol and total ammonia levels, combined with variations in transepithelial potential^29^. Interestingly, other studies documenting the physiological response of fish to acid stress^30^ show that fishes acclimate to the acid environment by modulating innate physiological parameters. Because innate physiological parameters are known to modulate both microbiota composition and activity^31^, the taxonomic structure of the endogenous microbiota on fish exposed to acidic pH will likewise be affected by the physiological response of its host. Perturbation of the taxonomic structure might elicit a disturbance of the bacterial functional repertory, impacting host immune defence. In addition to the host-associated physiological perturbation effects, there are potentially direct effects of the pH drop on microbiota as well, which can work synergistically with the host-associated perturbation. However, direct effects of pH drop and changes associated to host acclimatization to acidic environment are inextricable from each other. This phenomenon is best explained by the host-microbiota interdependency.

We hypothesized that exposure to short-term acid stress triggers a perturbation of the taxonomic structure of commensal microbiota (dysbiosis), potentially followed by a replacement with opportunistic bacteria from the environmental water or rare endogenous strains that are more resistant to acidic conditions. Based on Wood and co-workers^29^ we also hypothesized that the dysbiosis is a transitory state, eventually followed by microbiota homeostasis recovery (i.e. resilience of the microbiota (human microbiota resilience are reviewed in Lozupone *et al*.^32^)). Most importantly, a differential response of the microbiota from different ecological niches (e.g. different body sites, like cutaneous mucus versus intestines) was expected since different body sites exhibit a unique physiological response to the acid stress^30^. Overall, the impact of acidic stress on the taxonomic structure of the endogenous microbiota on teleosts has not been documented yet.

To address these questions, 72 juveniles of *Colossoma macropomum* were randomly distributed across a system of six independent tanks, and half of them were then exposed to acid stress (pH 4.0) during two consecutive weeks. Cutaneous mucus, feces and tank water were sampled at three sampling times: day 0, after one week and two weeks exposure to acidic water. Taxonomic structure of microbial communities was characterized by targeting the V4 region of the 16S gene.

## Results

A total of 6 911 554 reads, assigned to 1607 OTUs with 97% identity threshold were obtained from the 85 samples. The OTUs were assigned to 525 genera, 190 families, 81 orders, 38 class and 23 phyla. OTUs with fewer than 100 reads or that only occurred in a single sample were filtered out as a step to improve accuracy and diversity^33^ After filtration, there were 6 072 125 reads left, assigned to 508 OTUs, with a mean of 279 OTUs per sample and an average of 77 302 reads per sample. The average Good’s coverage estimation, used to assess the sampling effort, is 98 ± 5% of estimated coverage.

### Impact of pH drop on taxonomic structure

**Figure 1:**
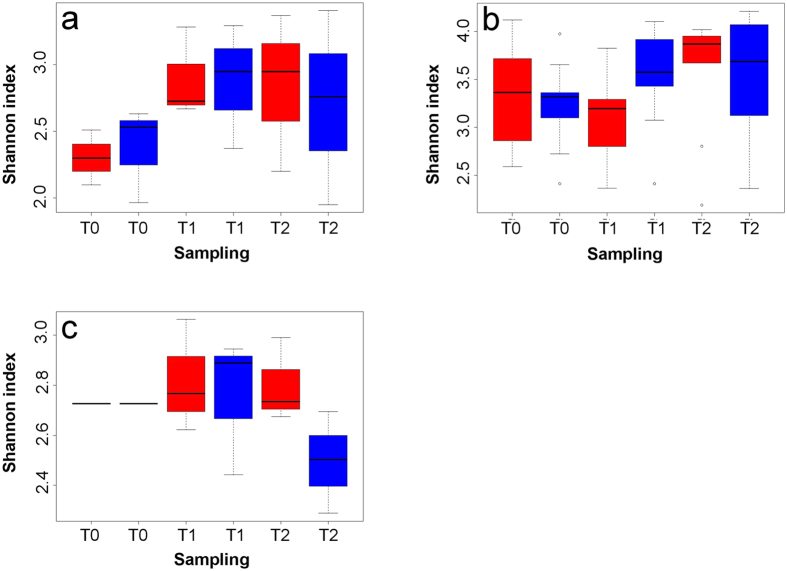
No significant difference of alpha diversity between sampling times, for all three ecological niches tested. The alpha diversity of bacterial communities at different sampling times is measured by non-parametric Shannon index of (**a**) feces samples, (**b**) skin mucus samples and (**c**) water samples. Boxes in red are test samples and in blue are control samples.

Non-parametric Shannon diversity indices for skin mucus samples showed the highest diversity (3.4 ± 0.2), which was significantly different from the diversity observed in feces (2.7 ± 0.2, p < 0.0001, df = 34, T = 5.39) and water samples (2.7 ± 0.1, p < 0.0001, df = 70, T = 7.66). There was no significant difference (p = 0.64, df = 22, T = 0.47) between diversity in feces and water samples. Finally, there was no significant variation between any sample categories (e.g. Control T0, Test T0, etc.) of the same sample type (e.g. water, feces or skin) in terms of Shannon diversity (Fig.1). Analysis of variance with permutations (PERMANOVA, 10 000 permutations^34^) (Table 1) revealed that the *treatment* factor (e.g. control or acid treatment) had a significant effect on the taxonomic structure found on the three microbial niches tested (water, feces and skin) (Table 1). At T1 (one week exposure to acid pH), the effect of the treatment was only significant in skin (p < 0.0001, R2 = 0.64) and feces samples (p < 0.002, R2 = 0.50). Although the treatment effect was still significantly different at T2 (two weeks exposure) for skin samples (p < 0.05, R2 = 0.57), it was not significantly different anymore for fecal samples (p > 0.05, R2 = 0.22). For water samples, the effect of the acid treatment was only significantly different at T2 (p < 0.002, R2 = 0.76). The *tank* factor showed significant difference only in skin samples at T0 and T1 (p < 0.05, R2 = 0.51). There was no significant difference between biological replicates.

**Figure 2:**
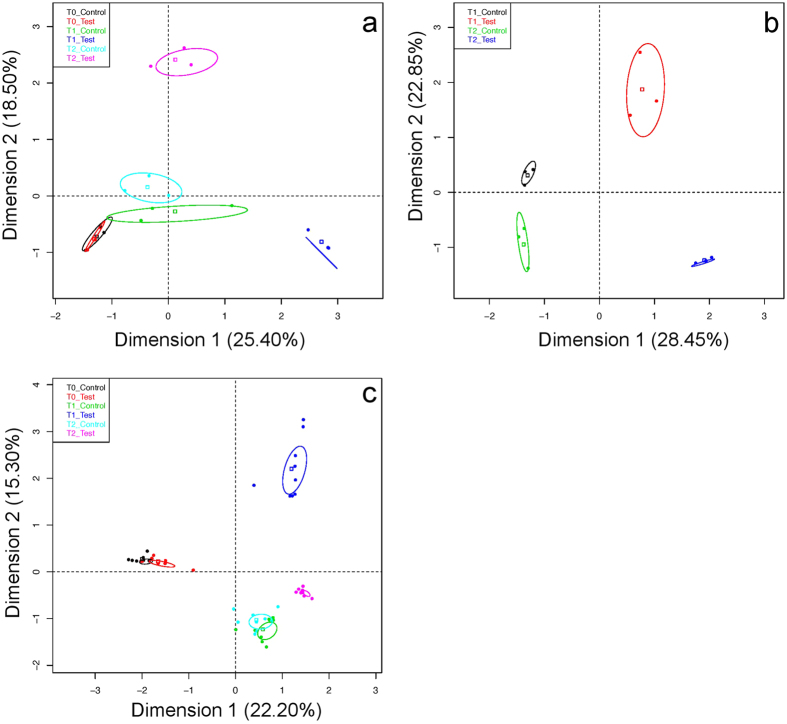
The taxonomic structure in all three niches is perturbed by pH drop. The multi-factorial analysis (MFA) based on the variations of relative abundance of bacterial genera in regard of the combination of the: “Sampling time” (T0, T1, T2) and “Treatment” (test or control) variables. (**a**) feces, (**b**) water and (**c**) skin mucus samples.

**Figure 3:**
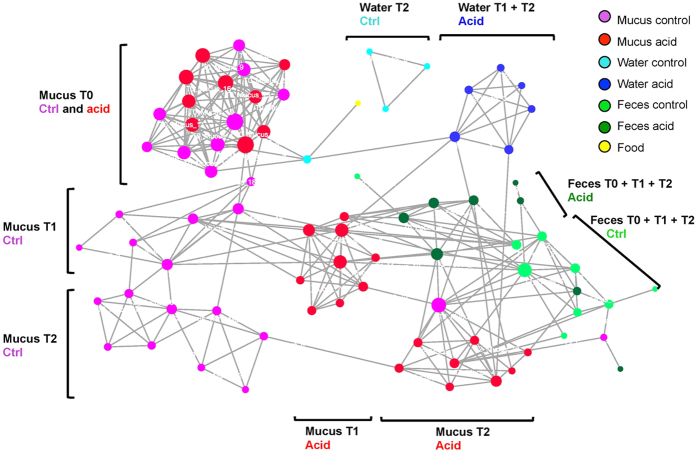
Taxonomic structures diverge between sampling times and ecological niches. The network of samples based on co-abundance of bacterial genera in each sample. Each dot (node) represents a sample. A link (edge) between two samples highlights a Spearman correlation index > 0.6 between the two samples and a p-value corrected with Bonferroni < 0.05. The size of each node is proportional to the number of edge that it is connected to.

The multiple factor analysis, like the analysis of variance, revealed that *acid treatment* had a strong effect on the taxonomic structure of the microbiota (Fig. 2). The first two axes of the graphical representation explain 37.5% of the variance in skin samples, 43.9% in feces and 51.3% in water samples. For the three niches tested, taxonomic structure of all *control* samples at T0, T1 and T2 was significantly different (p < 0.05) from the structure observed in T1 and T2 *test* samples. Note that there is no T0 water sample in the graphical representation, as only one sample was taken at T0 for this niche (tanks were connected until T0). *Control* samples at T1 and T2 are significantly different (p < 0.05) for feces and water, although they are more similar than *test* samples at T1 and T2. Based on the number of edges (links) between samples and overall layout of sample clusters, the co-abundance network analysis (Fig. 3) showed that the taxonomic structure in all samples was mostly affected, in decreasing proportions of variance explained, by (1) the *ecological niche* (water, feces or skin); (2) the *sampling time* (T0, T1 or T2); and (3) the *treatment* (control or acid). The network revealed a strong inter-niche differentiation in core taxonomic structures. The layout of samples showed that pH drop impacted differentially skin and gut microbiota taxonomic structures. All skin samples clustered closely together at T0, then the network showed a strong differentiation at T1 and T2 between *control* and *test* skin samples. In this respect, there were first 59 edges (i.e. significant Spearman correlations between taxa, Bonferroni corrected p-value < 0.05) at T0 between *test* and *control* skin, then the number of edges dropped to ten edges at T1, and only one at T2. Also, there was no significant Spearman correlation (Bonferroni corrected p-value > 0.05) between T0 and T1 *test* skin samples. The network revealed no significant correlation between the food sample (Purina pellets) and fecal samples. As shown in the PERMANOVA (Table 1), the abundance of edges (significant Spearman correlations) in the network between skin samples at T0 confirms that biological replicates exhibited a high similarity in terms of taxonomic structure.

### Acid exposure driven taxonomic shifts

**Figure 4:**
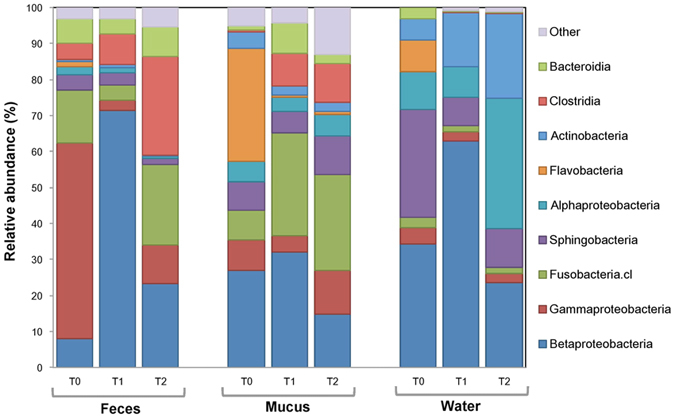
There are major taxonomic structure shifts at the class level after acidic water exposure. Variations of relative abundance (%) of 10 most abundant bacterial classes over sampling time and ecological niche.

Figure 4 shows that the relative abundance of the 10 most abundant bacterial classes differed between each sampling time and ecological niche in bacterial communities exposed to acidic treatment. Interestingly, the most notable taxonomic shift is the increase of the relative abundance of the class *Betaproteobacteria* at T1 compared to T0 (p = 0.014) and T2 (p = 0.047) in fecal samples. At the genus level, in the tanks receiving the acid treatment, *Flavobacterium* composed 31.0% of the skin mucus community and 9.0% of the water community at T0. At T2, these relative abundances respectively decreased to 0.75% and 0.03%. The significant reduction of the relative abundance of *Flavobacterium* between T0 and T1, was 11.2% higher in *test* skin samples than in *control* (p < 0.0001) and 37.2% higher in *test* water samples than in *control* (p < 0.002).

In *control* tanks, the genus *Undibacterium* composed a low average of 1.48% of the microbial community in feces, 1.38% in skin and 0.08% in water. In *test* tanks, between T0 and T1, the relative abundance of the genus *Undibacterium* increased by 1652% (reaching an abundance of 14.4%) in skin samples (p < 0.01) and by 273% in fecal samples (p < 0.01). In *test* water samples, this genus increased from 0 detected reads (T0) to a relative abundance of 3.7% of the microbial community at T1. In *test* fecal samples, the relative abundance of *Undibacterium* further increased by 229% between T1 and T2, then reaching a relative abundance of 7.35%. For *test* skin samples the relative abundance of this genus decreased of 78% between T1 and T2.

In *control* tanks, the genus *Duganella* composed an average of 5.38% of the microbial community in feces, 0.53% in skin and 0.26% in water. In *test* tanks, between T0 and T1, the relative abundance of this genus increased significantly (p < 0.001) by 4583% (reaching a relative abundance of 21.6%) in fecal samples. In *test* water samples, this genus increased from 0 reads (T0) to a relative abundance of 17.11% at T1. Between T1 and T2 ( >1 week after the initial pH drop in *test* tanks) there was a significant decrease in the relative abundance of *Duganella* both in water (−96.6%, p < 0.01) and feces (−56.1%, p < 0.02).

## Discussion

The results showed unambiguously that the acid pH drop had a significant effect on the structure of the bacterial communities that are closely associated with *Colossoma macropomum*. Structural taxonomic changes triggered by the pH occurred *in all of the three microbial niches* closely associated with *Colosoma macropumum*: surrounding water, intestine and epithelial mucus. As described on *Salvelinus fontinalis*^16^, one of the first perceivable changes on the microbiota after stress exposure is a taxonomic structure perturbation. This perturbation can act as a precursor to dysbiosis, leading to an imbalance in the equilibrium between opportunistic strains and pathogens^15^,^16^.

Each of the three microbial niches closely associated with *Colosoma macropumum* had a **specific response** to the acidic exposure. After one week of acidic exposure, there was a **significant change in taxonomic structure in host-associated microbiota** (i.e. epithelial mucus and fecal samples). Environmental pH has been recognized as a major driver of bacterial diversity in soil^35^. To add to this effect, physiological acclimatization of the host (gill structure breakdown, massive mucus production, cortisol synthesis)^16^,^29^ could have further increased the effect of acid stress on endogenous bacterial communities that are affected by (1) acid stress exposure combined with (2) a variation of available resources for growth on the host. Contrastingly, no significant change in taxonomic structure was observed in bacterioplankton at T1, highlighting the documented differentiation between host-associated microbial niches and the surrounding water^36^. However, we cannot rule out the possibility that important daily water changes (50% of tank volume) could have mitigated the observed perturbation on this particular niche, although pH was immediately adjusted to the target value.

**No significant difference was detected in alpha diversity** between control and acid exposed samples (Fig. 1). Pennanen and co-workers^37^ obtained similar results after exposing soil bacterial communities to simulated acid rains, suggesting that stress exposure triggers a microbial community succession (i.e a replacement of abundant taxa sensitive to acid pH by initially rare and more tolerant taxa). The microbial succession observed allowed us to detect **potential stress specific biomarkers**. Biomarkers (a substance produced by a living organism, or a living organism itself) are powerful and insightful tools to assess the biological state (e.g. health or perturbation) of an ecosystem^38^ or of a holobiont. Bacterial biomarkers in general are promising because of the short generation time of bacteria and their high sensitivity to variations in their environment making them a fast and precise tool to detect the effect of environmental perturbation on holobionts. Our results suggest that the class *Betaproteobacteria*, as well as the genera *Flavobacterium*, *Duganella* and *Undibacterium* have a great potential to be used as **stress specific** taxonomic biomarkers. The results revealed an increase in the relative abundance of the phylum *Proteobacteria* after acid stress exposure. The increase in the class *Betaproteobacteria* is significant (p < 0.05) in the three niche sampled in *test* tanks at T1. At T2, the relative abundance of this class significantly decreased (p < 0.05) from T1. These results further highlight the opportunistic nature of *Proteobacteria*, which was documented by Shin and co-workers (2015)^39^ and by Laplante and co-workers (2013)^40^ in acid mine drainages. Important variations were also observed for *Flavobacterium*, *Undibacterium* and *Duganella* genera. The genus *Flavobacterium* demonstrated a high sensitivity to acid pH. This is consistent with the results obtained by Suomalainen and co-workers (2005)^41^, who tested the effect of acid baths on rainbow trout against the opportunist pathogen *Flavobacterium columnare*. Results from our experiment suggest that in the future, acid baths could be a potential approach to decrease *flavobacteriosis* infections in tambaqui aquaculture systems, as an efficient alternative to antibiotic treatment or vaccination. On the opposite, *Undibacterium* and *Duganella* exhibited a different response than *Flavobacterium* to the acidic exposure: their relative abundance increased significantly the day following the pH drop (T1) and decreased significantly after stabilization of the pH (T2). These observations suggest that these taxa thrive better in perturbed or extreme environments. Interestingly, strains of *Undibacterium* have been documented in arsenic contaminated ground water^42^ and *Duganella* have been found in ground water beneath a uranium mine^43^.

The PERMANOVA reveals that **fecal microbiome community structure is more resilient than cutaneous microbiota** (see sampling T2 in Table 1). Gut microbiota are naturally exposed to acidic pH from gastric secretions; Payne (1978)^44^ has documented gastric pH as acid as 1.0 on the tilapia *Tilapia guineensis*. On the same species, the intestinal pH is circumneutral, highlighting the efficiency of pH buffering by alkaline secretions in the lower GI tract. Thus, given the efficiency of the intestinal pH buffering, it is possible that the dysbiosis detected in our study at T1 on fecal samples did not result directly from acute acidic exposure, but rather from host physiological stress. On the contrary, skin mucus communities likely encountered acute and prolonged acidic exposure after pH drop. Persistent dysbiosis state observed at T2 in skin mucus samples and water samples, but not in fecal samples, highlights this presumption. We cannot rule out the possibility that the taxonomic structure of the bacterial communities in skin mucus and water samples would stabilize over time. However, multiple evidence suggest that under a permanent exposure, it is unlikely that the taxonomic structure of these two particular niches will recover a taxonomic composition similar to the control group. Boutin *et al*.^16^ have shown that there was no significant resilience of the skin mucus bacterial community after a short exposition to hypoxia followed by a recovery time of 4 weeks. Laplante *et al*.^40^ have shown that there was no resilience for the water bacterial community in 5 lakes exposed to a gradient of polymetallic perturbation after a long-term exposition of 60 years.

Our results show that tambaqui exposed to acid treatment had a significantly higher ratio *Firmicutes* - *Bacteroidetes* in fecal samples than in control groups. In humans, many studies documented that a high *Firmicutes* / *Bacteroidetes* ratio could be significantly linked with obesity^45^. In fish models, Li *et al*. (2013)^46^ have showed that a high *Firmicutes* / *Bacteroidetes* ratio could be linked to faster growth rates of the transgenic common carp (*Cyprinus carpio* L.). To our knowledge, this is the first evidence that exposure to acidified water is linked to a higher *Firmicutes* - *Bacteroidetes* ratio. A long-term study needs to be exercised to assess if this ratio significantly affects the overall growth of *Colosoma macropumum* in an aquaculture system, as this could provide useful insight for tambaqui fish farmers in optimizing tambaqui growth rate.

The microbial community succession observed on all three ecological niches after exposure to environmental perturbation highlights the **adaptation capacity of the endogenous microbial communities**. As a host acclimatizes in response to a physiological stress, microbial communities at the interface between the host and the environment rapidly adapt to the modified ecosystem^40^,^47^. Interestingly, Rolli *et al*. (2015)^48^ have also shown that bacteria of root-associated microbiome can contribute to improve the adaptation of the holobiont to particular environmental conditions. Thus, it would be of great interest to assess the potential role of teleost-associated bacterial communities in facilitation of host-acclimatization to environmental stress. Indeed, as a host organism with great phenotypic plasticity might be adapted to a wide range of environmental conditions, an endogenous microbial community with substantial meta-genotypic plasticity (in this case, genotypic plasticity refers to the adaptability of the bacterial functions repertory) might confer to the holobiont a greater tolerance to fluctuating environments.

Overall, our results have shown that exposition of tambaqui to acid pH drop triggered dysbiosis in the microbiota of the three microbial niches investigated: cutaneous mucus, feces and environmental water. Furthermore, each microbial niche tested responded specifically to the acidic exposure. In this respect, our results highlight the strong resilience of tambaqui’s gut microbiota, which may be linked to its capacity to migrate from white water to black water. We have identified four potential stress specific taxonomic microbial biomarkers to assess the effect of environmental perturbation on aquatic biota: the class *Betaproteobacteria*, as well as the genera *Flavobacterium*, *Duganella* and *Undibacterium*. The results also revealed that acidic exposure had two potentially beneficial effects for tambaqui’s aquaculture: (1) a significant decrease in the relative abundance of the genus *Flavobacterium* responsible for *flavobacteriosis* infections and (2) a significant increase of the ratio *Firmicutes* - *Bacteroidetes* in feces. A long-term study is necessary to assess if the increase of this ratio improves overall growth of *Colosoma macropumum*. In the near future, a metagenomic approach is needed to assess whether the taxonomic structure perturbation of the tambaqui’s microbiota translates into an impairment of the microbial functional repertories dedicated to maintain the homeostasis of host physiological functions.

## Methods

### Fish rearing

The fish were reared in six independent 310 L FortLev tanks (3 control and 3 acid treatment tanks) at the “Laboratório de Ecofisiologia e Evolução Molecular” (LEEM) at “Instituto Nacional de Pesquisas da Amazonia” (INPA) in Manaus. Each tank contained 12 juvenile specimens of tambaqui (N_control_ = 36, N_test_ = 36, N_total_ = 72) obtained from a local fish culture station (average weight ≈40 g and length ≈15.0 cm). All of the fish were acclimated in the same conditions during six weeks before the start of the experiment: 12 h photoperiod, [dissolved O_2_] 7.1 ± 0.9 mg/L, water temperature 27.6 ± 0.4 °C and conductivity 48 ± 11 μmhos/cm. Every day, tanks were siphoned and 50% of the water was replaced by soft water from a local well with the following water parameters: [Ca^2+^] 11 μmol L^−1^, [Na^+^] 34 μmol L^−1^, [Cl^−^] 28 μmol L^−1^, [Mg^2+^] 0,8 μmol L^−1^, [K^+^] 15 μmol L^−1^, [dissolved organic matter] 0,9 mg C L^−1^, pH 6.3 and [NO_2_] 0–0,5 ppm. Fish were fed once per day to satiation with Purina^**®**^ commercial pellets. Each fish was tagged with injection of Visible Implant Elastomer tags (Northwest Marine Technology’s) (colors = pink, green, blue) on the dorsal fin. Four fish per tank were tagged with the same color (3 colors per tank in 6 tanks, N = 18 fish groups).

### Ethics statement

This project and protocol were approved by the Ethics Committee for the Use of Animals of INPA (number 026/2015 as of Dec 18th, 2015). All methods were carried out in accordance with the approved guidelines.

### Sampling

Skin mucus, feces and tank water were sampled (1) after the acclimatization period at a stable pH of 6.3 (T0 = day 1); (2) after a slow pH drop from 6.3 to 4.0 (T1 = day 7) and (3) after one week exposure to pH 4.0 (T2 = day 14). The pH in experimental tanks was adjusted gradually from 6.3 to 4.0 using diluted HNO_3_ 1 M and pH was stabilized at 6.3 in control tanks using (if needed) diluted HNO_3_ 1 M and KOH 1 M. **Skin mucus** sampling was performed by gently rubbing a sterile cotton swab on ≈30% of the total surface of the right side of each fish. Skin mucus samples were pooled per tank and per color group (pink, green, blue) and were stored at −80 °C in 1.5 mL Eppendorf tubes. **Feces** were siphoned at the bottom of each tank. All feces samples were pooled per tank and stored at −80 °C in 1.5 mL Eppendorf tubes. Since numerous studies suggest that components of fish microbiota are shared with the bacterial community in the surrounding water^21^,^49^,^50^, 500 mL of tank water was sampled. **Water** samples were collected in each tank at T1, T2 and one water sample was collected for the whole system at T0. Water samples were filtered on 0.2 μm membranes (Nucleopore^**©**^) using a vacuum pump. Post-filtration, the membranes were stored at −80 °C.

### Sample processing

DNA extraction of skin mucus samples and 0.2 μm membranes from water samples was performed using DNeasy^®^ Blood and Tissue Kit from QIAGEN according to the manufacturer’s instructions. DNA extraction of feces samples was performed using QIamp^®^ Fast DNA Stool Mini Kit according to the manufacturer’s instructions. Extracted DNA from feces, skin mucus and water was stored at −80 °C until amplification. DNA was amplified using universal primers specific to region V4 of the rRNA 16S gene. The hypervariable region V4 is often used to characterize thoroughly vertebrate-associated microbiota^49^. The forward primer S-D-Arch-0519-a-S-15 of sequence 5′CAGCMGCCGCGGTAA-3′ and the reverse primer S-D-Bact-0785-b-A-18 of sequence 5′-TACNVGGGTATCTAATCC-3′ were used. PCR reactions were performed according to the manufacturer instructions of TaKaRa Premix^™^ 2.0. Each reaction tube contained 5 μL of TaKaRa Premix^™^ 2.0 and 1 μL of each primer. Volume was completed to 11 μL with QIAGEN’s Microbial DNA Free Water and template DNA. PCR were done in triplicates to reduce PCR bias. PCR program: (1) 10 min 94 °C; (2) 1 min 94 °C; (3) 1 min 55 °C; (4) 1 min 30 sec 72 °C; (5) 10 min at 72 °C; 30 amplification cycles total. The 3 reaction replicates per sample were merged in an Eppendorf 1.5 mL post-PCR. Amplified DNA was purified with AMPure beads (Beckman Coulter Genomics) to eliminate primers, dimers, proteins and phenols. 50 μL of Microbial DNA Free Water and 10 μL of beads were added to each sample. Samples were mixed manually and incubated 5 min at RT. Using a magnetic particle concentrator (MPC), beads were concentrated on the side of the Eppendorfs and supernatant was removed. Beads were washed two times with 500 μL ethanol 70% and incubated 30 sec at RT during each wash. Supernatant was removed and beads were left to dry 5 min. Tubes were removed from the MPC and 20 μL of Microbial DNA Free Water were added for elution. DNA concentration and quality were assessed by electrophoresis on 1.5% agarose gel and by NanoDrop. After purification, samples were sequenced in the MiSeq platform from Illumina, at the LEEM. The Nextera^**®**^ XT DNA Library Preparation Kit (96 samples) (Catalog number: FC-131-1096) was used for DNA library preparation. The Illumina^**®**^ MiSeq Reagent Kit v2 (500-cycle; 2 × 250) (Catalog Number: MS-102-2003) was used for sequencing on MiSeq (Illumina Sequencing System). All proceedings followed the manufacturer’s protocols.

### 16S amplicon sequences analysis

The sequence files are available from the Sequence Read Archive (http://www.ncbi.nlm.nih.gov/sra), *BioProject ID*: PRJNA323592. The analysis of amplicon sequences was done at the Institut de Biologie Intégrative et des Systèmes (IBIS) at Université Laval. Quality trimming of the sequences was done with sickle^50^. Then, the paired-end Illumina reads were merged with pandaseq^51^. Mothur alignment function^52^ was performed to align each read to 16S V4 sequence as reference template. Uchime^53^ was used to remove chimeric sequences. The clustering software Usearch^54^ was used for de novo operational taxonomic unit (OTU) picking with an identity-clustering threshold of 97%. RDP database reference taxonomy files were used to annotate OTUs^55^. Given the variability of the V4 region^56^, a bacterial genus was assigned to each OTU. OTUs with ≥100 reads total in all samples were kept for downstream analysis. Alpha diversity measures were obtained with mothur software^52^. Home made PERL scripts were developed to annotate OTUs taxonomy labels and to adapt the OTU table to the format requirements of mothur. The normalized OTU table was used for all analysis except for alpha diversity and Good’s index.

Shannon’s non-parametric alpha diversity index and Good’s coverage index were assessed for each sample, using mothur software^52^. Variance equality was assessed with F-tests on StatPlus v.5.9.92. T-tests for two-tailed distribution with unequal variance were used for non-parametric Shannon diversity indexes. Alpha diversity (see Fig. 1) was then represented in boxplots constructed with R version 3.2.1. A permutational analysis of variance (PERMANOVA) was performed with R to assess the effects of (1) the acid treatment; (2) the inter-tank variations; (3) the pooled samples (biological replicates); and (4) the time, on the variation in the taxonomic structure of the bacterial communities between samples. For each analysis of variance, 10 000 permutations were applied. To visualize the degree of similarity between samples taxonomic structures, a network of samples, based on OTU relative co-abundance between samples, was constructed with Cytoscape version 3.2.1^57^. To construct this network, the Spearman’s correlation was calculated between each sample pair using R. A p-value, to which was applied Bonferroni correction, was determined for each Spearman’s correlation value. The significant correlation values used for the network construction had a Spearman’s correlation ≥ 0.6 and a Bonferroni corrected p-value ≤ 0.05. The nodes of the network represent the samples and the edges (i.e. connection) are attributed to significant correlation between nodes. To confirm the results obtained with PERMANOVA and the Cytoscape network, a Multiple Factor Analysis (MFA) was done with R. The MFA is similar to a Principal Components Analysis in data multidimensional visualization and it was used to simultaneously study global patterns observed in continuous (OTU normalized abundance) and categorical (e.g. time of sampling) variables. Confidence ellipses were drawn (with default settings of the *plotellipses* function) around each category (e.g. Control T0, Test T0, etc.) in the graphical representation. Categories with non-overlapping ellipses were considered significantly different (default confidence level of 0.95).

## Additional Information

**How to cite this article**: Sylvain, F.-É. *et al*. pH drop impacts differentially skin and gut microbiota of the Amazonian fish tambaqui (*Colossoma macropomum*). *Sci. Rep.* **6**, 32032; doi: 10.1038/srep32032 (2016).

## Figures and Tables

**Table 1:** The differential response of exposure to acid pH is niche-dependant.

Sampling	Variable	*p*-value Skin mucus	*p*-value Feces	*p*-value Water
T0	Biological replicate	1	N/A	N/A
	Tank	0.0441*	1	N/A
	Treatment	0.1623	0.4	N/A
T1	Biological replicate	1	N/A	N/A
	Tank	0.0002***	1	1
	Treatment	0.00009999***	0.001389**	0.1
T2	Biological replicate	1	N/A	N/A
	Tank	0.1147	1	1
	Treatment	0.0494*	0.3014	0.001389**

P-values from PERMANOVA highlight the effect of the variables: “Biological replicate” (groups of 4 fish in the same tank), “Tank” and “Treatment” (acid stress or control) on bacterial communities in three ecological niches (skin mucus, feces and water) at three sampling times. P-values in green are < 0.05. No data is available for feces and water samples for biological replicate, because only one sample was taken in each tank. No data is available for water at T0 because only one sample was taken for the whole system.
